# Chatbot for the Return of Positive Genetic Screening Results for Hereditary Cancer Syndromes: a Prompt Engineering Study

**DOI:** 10.21203/rs.3.rs-4986527/v1

**Published:** 2024-08-29

**Authors:** Emma Coen, Guilherme Del Fiol, Kimberly A. Kaphingst, Emerson Borsato, Jackie Shannon, Hadley Stevens Smith, Aaron Masino, Caitlin G. Allen

**Affiliations:** Clemson University; University of Utah; University of Utah; University of Utah; Medical University of South Carolina

**Keywords:** Prompt Engineering, Few-Shot Learning, Retrieval-Augmented Generation (RAG), Population Screening Program

## Abstract

**Background:**

The growing demand for genomic testing and limited access to experts necessitate innovative service models. While chatbots have shown promise in supporting genomic services like pre-test counseling, their use in returning positive genetic results, especially using the more recent large language models (LLMs) remains unexplored.

**Objective:**

This study reports the prompt engineering process and intrinsic evaluation of the LLM component of a chatbot designed to support returning positive population-wide genomic screening results.

**Methods:**

We used a three-step prompt engineering process, including Retrieval-Augmented Generation (RAG) and few-shot techniques to develop an open-response chatbot. This was then evaluated using two hypothetical scenarios, with experts rating its performance using a 5-point Likert scale across eight criteria: tone, clarity, program accuracy, domain accuracy, robustness, efficiency, boundaries, and usability.

**Results:**

The chatbot achieved an overall score of 3.88 out of 5 across all criteria and scenarios. The highest ratings were in Tone (4.25), Usability (4.25), and Boundary management (4.0), followed by Efficiency (3.88), Clarity and Robustness (3.81), and Domain Accuracy (3.63). The lowest-rated criterion was Program Accuracy, which scored 3.25.

**Discussion:**

The LLM handled open-ended queries and maintained boundaries, while the lower Program Accuracy rating indicates areas for improvement. Future work will focus on refining prompts, expanding evaluations, and exploring optimal hybrid chatbot designs that integrate LLM components with rule-based chatbot components to enhance genomic service delivery.

## Introduction

The increased demand for genomic testing, resulting growth in patient volume, and limited access to providers with genomic expertise has necessitated new, innovative genetic service delivery models.^1–6^ Prior research has demonstrated the feasibility and acceptability of incorporating technologies such as chatbots to support common communication that occurs throughout the genomic service delivery process.^7–10^ Chatbots are a highly accessible and scalable platform that allow for simulated conversations. Accessible via Web through a hyperlink or downloadable app, chatbots can be used on a smartphone, tablet, or computer. The use of chatbots has also been shown to improve access to services and support health equity by providing personalized health education, being available in multiple languages, and offering continuous access to information.^11–15^

The integration of chatbots into routine and ancillary tasks such as pre-test counseling education, informed consent, delivery of negative results, and cascade testing have been shown to be feasible and effective in supporting genomic service delivery.^8,16^ For example, chatbots have been used to collect family health history, to provide pre-test support, communicate with family members about results, and obtain consent for genomic research.^8,17–19^ Prior results from the Broadening the Reach, Impact, and Delivery of Genetic Services (BRIDGE) trial showed equivalence between a technology-based chatbot approach and standard of care in completion of pre-test genetics education and completion of genetic testing among unaffected primary care patients meeting criteria for cancer genetic evaluation (In Press). Additional research in other health service delivery contexts has found that patients using chatbots reported better understanding about their condition or procedure, being more prepared for upcoming appointments, and feeling more informed when making health care decisions.^20–28^

To date, the integration of chatbot technology into genomic service delivery has yet to focus on the return of positive genetic test results directly to patients. Currently, return of positive results has been carried out largely through direct communication, due to the complex and sensitive nature of the information, potential psychological impact of learning about genetic predisposition, and need to ensure understanding of the results and their implications. However, non-chatbot technology-based solutions, such as online patient portals, are available to communicate with patients about these results and have been shown to be highly acceptable and preferred in genomics research.^8,10,16,29–34^ Furthermore, a large-scale study across three academic medical centers found that individuals preferred laboratory test results be delivered immediately online.^29^

Prior qualitative data has indicated patients are favorable toward receiving results via chatbots, as they are convenient and allow for the opportunity to contemplate information and ask questions.^8^ Digital health communication approaches, such as chatbots, may be especially appropriate for disclosure of population-based genomic screening (PGS) results. PGS is often conducted on a large scale, targeting asymptomatic individuals as part of public health initiatives. As a result, the communication typically emphasizes general risk awareness, with initial results disclosure indicating increased risk rather than confirming a diagnosis. The Consent and Disclosure of Recommendations (CADRe) workgroup funded by the National Cancer Institute’s Clinical Genome Resource (ClinGen) recommends considering factors such as test complexity, testing situation complexity, implications of genetic diagnosis to the patient and family, evidence of potential adverse psychological impact, and availability of high-quality and patient friendly materials when deciding on the level of interaction with the patient.^35,36^ Since PGS is typically completed through research and consent from participants and individuals are receiving results for well-defined hereditary conditions, the necessary level of initial communication about positive PGS results is lower than more complex, clinical results.

While high levels of acceptability, usability, and understanding of chatbots have been found in prior research, the majority of chatbots developed to date are rule-based, meaning that they operate on a set of pre-defined navigation paths with predefined scripted options and responses.^8,9,19^ This approach allows for reliability and consistency in managing response options. However, user testing of rule-based chatbots has also revealed a need for chatbots that allow users to ask open-ended questions and receive responses in real time.^8,9,19^ More recently, the release of large language models (LLM) such as ChatGPT offers an opportunity to direct open-ended questions to LLMs to better support return of positive genetic testing results, as open-ended questions allow for more nuanced and personalized responses. However, it is critical to test such systems to ensure that patients would receive accurate and clear information. Indeed, creating a hybrid chatbot with both rule-based and LLM components can offer a versatile and streamlined user experience by ensuring that key information is covered in the rule-based components of the chatbot and allowing for the LLM component to support complex, open-ended queries that are not covered in the scripted content. The objectives of the present study were 1) to prompt engineer an LLM-based chatbot focused on answering questions about return of positive PGS results; and 2) conduct an intrinsic evaluation of the prompt engineering approach based on hypothetical cases and expert raters.

## Methods

### Study Setting

We developed this chatbot for the context of answering questions about return of PGS results for an ongoing PGS program being delivered at the Medical University of South Carolina. The PGS program was established in November 2021 with the focus on providing free genetic screening to 100,000 individuals in South Carolina. To date, the program has recruited 59,352 individuals, returned 33,142 results and identified 132 individuals with Lynch Syndrome, 265 with HBOC, and 191 for FH.

### Prompt Engineering Approach for Open-Ended Content

LLM models have been applied to improve accuracy and standardization for a variety of biomedical tasks including medical guidelines retrieval, diagnostics, medical reporting, and medical education.^37–39^ The LLM selected depends on the task at hand, with a variety of LLMs developed for specific medical tasks and specialties.^40^ Commonly used LLMs include ChatGPT, Perplexity AI, Claude AI, and Google Bard.^41^ Developing Generative AI standards emphasize the need to design generative AI tools responsibly for user mental models and build trust while allowing for generative variability, co-creation, and imperfection.^45^ Meeting these standards requires effective prompt engineering, which includes the process of developing the text that instructs the LLM to complete a given task.^46^

We used a three-step prompt engineering approach using the Retrieval-Augmented Generation (RAG) technique which integrates retrieval-based methods with generative models, enabling the generation of contextually informed responses by retrieving relevant knowledge from a large corpus and incorporating it into the output generation process. RAG has been shown to improve LLM model performances by incorporating external information as a domain specific knowledge base.^42,43^ This study used OpenAI’s GPT Version 4-Turbo-Preview model, as new research has indicated GPT version 4 performs significantly better at answering genetics questions than version 3.5.^44–46^ OpenAI’s Playground was used for prompt engineering and testing. GPT4 was trained to respond about a variety of topics including providing examples of the impact of positive results, screening recommendations, and family history and cascade testing resources, and providing details regarding genetic counseling and specific PGS programs. Boundaries were also provided to ensure GPT4 responses remained within the intended scope of the chatbot.

Step 1: Provide Content and Context to GPT4. We used the RAG technique for prompt development. The RAG approach consisted of providing supplementary materials that were uploaded through OpenAI’s Playground “File Search” function which allows GPT4 to access the additional information in real time when responding to users’ questions. The additional files uploaded were: 1. Detailed descriptions and FAQs from the Medical University of South Carolina (MUSC)’s PGS website, 2. MUSC Genetic Counseling Scripts: Standard scripts used by genetic counselors at MUSC, providing insights into professional communication and common queries, and 3. Genome Medical Genetic Counseling Scripts: scripts from Genome Medical to offer additional perspectives. These documents expanded the model’s knowledge base to ensure detailed, consistent, and accurate responses.

Step 2: Establish a Bank of Commonly Asked Questions. To train and test the LLM, a bank of commonly asked questions was developed. This bank of questions was derived from patient quality improvement interviews and expert input. This step ensures that the model is trained on a wide array of realistic and relevant scenarios, enabling it to provide accurate and helpful responses. The list of 27 questions was randomly divided in to 13 training questions and 14 evaluation questions (Supplemental File 2).

Step 3: Develop and Refine Prompts. The core of prompt engineering involves creating and refining prompts that train the AI model to elicit the most accurate and appropriate responses. The prompt development process used OpenAI GPT assistants to develop an initial draft prompt. The prompt aimed to not only inform the chatbot about the situational context and content to be discussed, but also about the writing style and limitations it should adhere to. We completed iterative testing by inputting the prompt as the instructions for the AI assistant and running the 13 training questions through the messaging feature. Adjustments were made to the initial prompt until the chatbot answers were deemed accurate, clear, and appropriate by our internal study team. The prompt indicated to the LLM that patient cases would be provided as input.

### Prompt Engineering Evaluation

After completing the prompt engineering of our LLM chatbot, we conducted an intrinsic evaluation based on two hypothetical cases that were presented to domain experts in clinical genomics. The evaluation consisted of two steps described below.

Step 1: Establish the Prompt Evaluation Criteria. Previous literature has indicated relevant criteria to consider for chatbots in health communication.^47^ Considering this previous work, we established relevant evaluation criteria tailored to this study through discussion and consensus among the study team (Table 2). Based on eight criteria, an evaluation instrument was developed in REDCap consisting of the eight criteria, their definitions, and the ability to rate each criteria using a 5-point Likert scale from “Very Poor” (1) to “Excellent” (5).

Step 2: Development of Case Scenarios and Expert Ratings. We developed two hypothetical scenarios focused on returning results to individuals whoparticipated in PGS. We used scenarios to allow the expert raters to view the chatbot from the perspective of a specifi c hypothetical patient (Table 3).

We provided the two case scenarios and the resulting script to the expert raters who were asked to rate the quality of the chatbot responses based on the designated criteria listed in Table 2. The evaluators were aware that the responses were generated by an LLM. Eight experts completed the evaluation of the LLM output for the two hypothetical scenarios. Experts included: two clinician-researchers with expertise in genomics, one genetic counselor, three program managers working with genomic screening programs, and two PhD-trained researchers with expertise in genomics. Descriptive statistics were calculated, including median and mean scores for each evaluation criterion.

## Results

### Final Prompt and Example Responses

The final prompt used for the LLM is included as Supplemental File 1. Instructions for the prompt included a description of the type of patients the chatbot would be interfacing with (e.g., patients who have done genetic testing as part of MUSC’s population genomics program and are receiving their results online), the types of questions the chatbot may receive (e.g., patient questions may range from understanding their specific probability of developing cancer to inquiries about insurance coverage), and boundaries (e.g., do not provide any kind of medical advice […] if the patient asks questions outside of your boundaries, reply with “I am unable to answer questions unrelated to genetic testing or helping you get to your first appointment with a genetic counselor.”). Other elements of the prompt included the style of the response and literacy level (e.g., you will be replying to adults, but use a fifth-grade level vocabulary. Provide a clear, direct, concise response, preferably in bullet point format). Examples of responses provided by the chatbot are included in Table 4.

### Prompt Engineering Evaluation

The expert ratings of chatbot responses are provided in Table 5. Overall, the chatbot received an average score of 3.86 across all criteria and cases. The ability to express information (tone) and ease of interface (usability) received the highest scores (4.25). Boundary was rated as 4.0 followed by efficiency (3.88). Clarity and robustness received scores of 3.81 followed by domain accuracy (3.63). The lowest rated domain was program accuracy 3.25.

## Discussion

We completed prompt engineering and intrinsic evaluation of the LLM component of a chatbot designed to facilitate the return of positive PGS results. Through RAG technique, we successfully developed a prompt tailored for this application. Eight experts performed an intrinsic evaluation, which assessed the chatbot’s responses to 14 questions across eight distinct domains in two hypothetical cases scenarios. The chatbot achieved an overall average score of 3.88 across all domains, with the highest ratings in the tone domain and the lowest in program accuracy. These findings will inform further refinement of the prompt and integration of the LLM with the existing rule-based system, ultimately leading to the development of a hybrid chatbot to support the return of genomic screening results.

### Domain Accuracy and Boundaries of LLM

Prior studies have indicated that individuals are favorable toward the use of chatbots for patient follow-up and genetic test results disclosure, with a preference to include open-ended response options.^8^ However, to date, few chatbots have incorporated LLMs to answer open-ended responses to questions about genetic testing in real time.^8,19^ LLM responses must be carefully engineered to ensure confidence in the accuracy and reliability of responses, as well as the ability to handle ambiguous questions.^48^ Our prompt engineering process resulted in a chatbot that performed well in the criteria of boundaries (ability to avoid answering questions that are unrelated to the topic), domain accuracy (ability of chatbot to provide correct information about the genetic test result and care implications) and robustness (ability to handle ambiguous queries or incomplete information). Another project focused on generative AI solutions for personalized pharmacogenomics recently identified similar trends. Murugan (2024) found that accuracy (degree to which the responses align with guidelines) of their chatbot was rated at 75th percentile and relevance (similar to our criteria of boundaries) was rated at the 78th percentile for patient-facing responses delivered by their chatbot.^49^ They significant differences in performance metrics for these domains across responses provided by ChatGPT 3.5 and their pharmacogenomics-specific AI assistant (71st percentile vs. 75th percentile for accuracy and 68th percentile vs. 78th percentile for relevancy), indicating the value in prompt engineering for specific use cases. Challenges exist in ensuring domain accuracy and boundaries, such as limitations in LLM’s context retrieval and ability to process specialized biomedical and genomic data.^50,51^

The combination of high domain accuracy and boundaries is essential for managing sensitive health information and mitigates concerns of chatbots offering misinformation and medical advice beyond the scope of the chatbot. As the LLM is further refined, it will be important to document all steps of the prompt engineering and be clear and transparent about the prompt engineering process used to develop the model in order to instill trust in the quality of responses and reduce the risk of misinformation.^48^ It will also be critical to involve patient stakeholders in the future evaluation process. Other approaches to prompt development and evaluation include involvement of experts (genetic counselors, oncologists) to help identify unintentional sources of bias and decide on high-quality data sources that can be used to train the model.^52^ Furthermore, given that the evaluation process included only a limited set of test questions, inclusion of a more comprehensive question set could provide additional insight into the chatbot performance and ensure its ability to manage a greater set of user interactions. For example, our testing included 14 questions, whereas other projects have included over 30 questions.^49^ In particular, future studies should incorporate adversarial examples in both engineering and testing, especially to more comprehensively test the model accuracy and boundaries.^52^

### Tone of Open-Ended Responses

In addition to domain accuracy and boundaries, it is critical to ensure open-ended, LLM-generated responses are delivered in a tone that instills trust and engagement with the individual. Expert ratings indicated that the chatbot had good quality tone (ability to express information in a way that is appropriate for the type of information being delivered), usability (ease of interfacing with the chatbot), efficiency (ability to answer in a way that is direct, concise, and complete), and clarity (ability to communicate information clearly and in a way that avoids confusion) in both case scenarios. Murugan (2024) assessed a similar domain of language and bias (clarity and neutrality of responses, ensuring the context is understandable and devoid of bias), which was rated highly (87th percentile).

While final prompt delivered relatively high quality responses in an appropriate tone; however, it is important to note that we did not assess perceptions of the quality of delivery among patients. Many chatbots have been designed to support mental health and behavior change modifications and are explicitly focused on building relationships and natural language experience for genomics-focused chatbots.^53,54^ Furthermore, we tested the responses for hypothetical scenarios returning Lynch Syndrome pathogenic variant (MLH1) and Hereditary Breast and Ovarian Cancer syndrome (BRCA) results. There may be a need to further refine and test response quality and tone across specific genes, as each has unique implications and thus may require distinct prompts. User testing among patients will also help address potential adaptations needed to ensure culturally appropriate responses.^55^

### Integration of LLM and Rule-Based Chatbot

Our long-term goal is to incorporate the LLM component of the chatbot described here with an existing rule-based chatbot called GRACE. This hybrid approach could be ideal for the return of positive PGS results, as it integrates scripted content that is critical for results disclosure with patient preference for open-ended response options. The combined approach can address the limitations of purely rule-based or purely LLM-driven systems to combine consistency and accuracy with conversational fluidity and content comprehensiveness. Some information may be more suitable to rule-based or scripted content. For example, in our intrinsic evaluation, the LLM chatbot received poor scores for program accuracy (ability of chatbot to provide correct information about the genomic screening program). Although provided materials about the specific program were included as part of prompt engineering, experts rated this lowest among the domains they evaluated. Indeed, this type of stagnant information does not require personalization and may be most suited for pre-scripted, educational content, whereas the LLM components are most suitable for complex and open-ended questions and more nuanced interactions.^48^

One hybrid approach could incorporate a scripted component that provides a pre-determined set of information, followed by a LLM component that is engineered specifically to support open-ended questions about a certain domain (Table 6). This may include key domains of: overview of the PGS program, returning positive results, screening recommendations, impact on family, and next steps.

Another hybrid approach could vary when the LLM or rule-based components are used throughout the chatbot. For example, the return of results process involves three main stages: engagement, activation, and addressing information needs. In the engagement stage, the rule-based component of the chatbot would provide an overview of the PGS program, inform the individual of their positive results and educate the individual about what this means for their long-term care. The activation phase could also use rule-based content and guide individuals through a core set of scripted information to encourage next steps. In the subsequent open-ended content, participants’ information needs could be addressed by allowing them to ask additional questions about topics they choose, which could be answered through the LLM.

### Strengths and Limitations

Our prompt engineering approach incorporated multiple techniques to develop a LLM chatbot that was well-rated across several quality domains. We used RAG as our approach to prompt development, but other techniques such as few shot, supervised fine-tuning and reinforcement learning from human feedback could be used to further adjust the model’s responses.^42^ In addition, we focus on a use case of returning positive results for PGS, as PGS results return is among the least complex type of results being disclosed and could benefit from incorporating automation. Limitations of the study include our small sample size for the intrinsic evaluation of the chatbot responses and lack of patients reviewing the responses. At this phase of the project, our goal was to develop the initial prompt and assess feasibility of the prompt to respond to questions about return of results. Thus, we did not include patients but will include patient perspectives and ratings of the quality of responses in future refinement of the LLM. Patients may identify areas for improvement that are not apparent to expert reviewers. Further, we only evaluate the script produced by LLM component of the chatbot across two use cases. Additional use cases should be assessed (e.g., other genes) to identify whether one prompt can be used or whether multiple prompts need to be developed for specific open-ended components of a hybrid chatbot. Finally, our assessment is only focused on the LLM component of the chatbot. Our future work will integrate the LLM component with the rule-based script, allowing us to assess different hybrid approaches. For example, we could address whether open-response options should be available as part of each component of the chatbot, which may require specific prompts for each component, or whether the open-response LLM component is generic.

## Conclusions

This study demonstrated initial feasibility of prompt engineering for the LLM component of a chatbot designed to return positive genomic screening results, with high expert ratings across most of the evaluation criteria. These preliminary findings will be used to further develop a hybrid chatbot that integrates the rule-based and LLM components to enhance the delivery of results by providing essential information with the flexibility of managing a range of patient queries. Further refinements of the prompt are needed, as well as broad user-testing that involves individuals with various genomic conditions and cultural preferences, and testing of the best integration of LLM and rule-based components of the chatbot. This new approach to conveying positive genetic screening results has promise and can help address the limitations of the current genomic workforce that would be needed for return of all positive results in a population genomic screening context.

## Figures and Tables

**Table 2:** Evaluation Criteria

Criteria	Quality Definition
Tone	Ability of chatbot to express information in a way that is appropriate for the type of information being delivered.
Clarity	Ability of chatbot to communicate information clearly and in a way that avoids ambiguity or confusion.
Program Accuracy	Ability of chatbot to provide correct information about the PGS program
Domain Accuracy	Ability of chatbot to provide correct information about the genetic test results and care implications
Robustness	Ability to handle ambiguous queries or incomplete information
Efficiency	Ability to provide answers that are direct, concise, and complete
Boundaries	Ability to avoid answering questions that are unrelated to the topic
Usability	Ease of interfacing with chatbot

**Table 3.** Case Scenarios

Case 1: Anna	Case 2: Josh
A 34-year-old White woman named Anna participated in the In Our DNA SC population-wide genomic screening program in November. She had no personal or family history of cancer. She saw an advertisement online and decided to participate in the program out of curiosity about her health. She had previously completed a direct-to-consumer genetic screen through 23 and Me but did not receive any concerning results. A few months passed after she provided her sample. Her results showed that she has a BRCA1 mutation. Anna was shocked to find this news about her BRCA1 mutation and is concerned about the next steps in her care	A 56-year-old Black man named Josh previously had colorectal cancer. His sister participated in In Our DNA SC based on a recommendation from her doctor. Josh’s sister convinced him that it would be beneficial for him to participate. He reluctantly agreed to participate but did not see much of a point since he already been treated for colorectal cancer. Josh received results showing that he was positive for the MLH1 gene, which means he has Lynch Syndrome. Josh was confused by these results. He did not understand how these results would be important to him since he already had cancer

**Table 4:** Sample Questions and LLM Responses

Question	LLM Response
What is the cost of genetic counseling visits and the screening?	I’m here to help, but remember, I can’t give you specific costs because they can vary a lot depending on several factors. Here’s what you should know about the costs of genetic counseling visits and screenings: • **Insurance Coverage**: Many health insurance plans cover genetic counseling and screening, especially if you have a higher risk for certain conditions. However, coverage can vary, so it’s important to check with your insurance provider. • **Genetic Counseling Costs**: The cost of a genetic counseling session can vary based on the location, the facility, and whether the counselor is part of a hospital or a private clinic. Some genetic counselors may work with you on payment options if you are paying out of pocket. • **Screening Costs**: Like counseling, the cost for genetic screenings can vary widely. It depends on the type of screening and whether it’s covered by insurance. • **Financial Assistance Programs**: Some hospitals, clinics, and organizations offer financial assistance or programs that might help cover the cost of genetic testing and counseling for those who qualify. It’s a good idea to: • **Contact your insurance company** to find out what they cover and if you need a referral. • **Ask the genetic counseling service** about costs and payment options when you schedule your appointment.
What is the recommended treatment/preventative care?	For individuals with an MLH1 mutation, recommended treatment and preventive care focus on lowering the risk of cancer and catching any signs of cancer early. While I can’t give personal medical advice, here are some general recommendations: • **Regular Screenings**: ○ **Colonoscopy**: Starting at a younger age and happening more often than for the general population, usually every 1 to 2 years. ○ **Physical exams**: Including checking for changes in your body that might indicate cancer. • **Lifestyle Changes**: o Eating a healthy diet. ○ Staying active. ○ Avoiding smoking. • **Family Planning Discussions**: Since this mutation can be passed to your children, talking with a genetic counselor about family planning options is helpful. • **Consider Surgery**: In some cases, to prevent cancer, doctors might suggest surgery to remove certain parts of the body that are at high risk of developing cancer. This is a personal decision and requires a detailed discussion with your healthcare team. • **Stay Informed**: New recommendations and treatments can become available. Keep in regular contact with your healthcare team. A genetic counselor can provide personalized information based on your specific situation and help you understand your options for prevention and early detection. It’s important to discuss the best plan for you with your healthcare provider or genetic counselor. Remember, these are general recommendations, and the best approach for you should be discussed with your healthcare provider. If you need help scheduling an appointment with a genetic counselor at the MUSC Hollings Cancer Center Hereditary Cancer Clinic, you can visit their website or call 843-792-0745 for more personalized support.
How can I stay up to date on the latest treatments and science about my pathogenic variant?	Staying informed about the latest treatments and science related to your MLH1 mutation is a great way to actively participate in your health care. Here are some ways to keep updated: • **Follow Reputable Sources**: Websites of major cancer centers, genetic organizations, and health institutions often share updates about new research and treatments. Some reliable sources include the National Cancer Institute (NCI), American Cancer Society (ACS), and the Genetic Alliance. • **Attend Appointments**: Regular check-ins with your healthcare team, including your genetic counselor, can keep you informed on any new recommendations or treatments relevant to your situation. • **Join Support Groups**: There are support groups and communities for individuals with hereditary cancer risk. These can be great places to share information and experiences. • **Subscribe to Newsletters**: Many organizations offer newsletters that summarize the latest research and developments in cancer treatment and genetics. • **Educational Events**: Workshops, webinars, and conferences designed for patients and families can provide valuable information and updates. • **Ask Questions**: Whenever you have an appointment with your healthcare provider or genetic counselor, ask if there are any new findings or treatments relevant to your mutation. Remember, while it’s great to stay informed, always discuss any information or potential treatments you come across with your healthcare provider before making any changes to your health care plan. They can help interpret how new information may apply to your specific situation. If you’re looking for specific resources or have more questions, feel free to ask.

**Table 5:** Expert Ratings for each Study Case and Combined

		Case 1: Anna				Case 2: Josh				Combined			
Quality	Quality Definition	Median	Mean	Standard Deviation	Range	Median	Mean	Standard Deviation	Range	Median	Mean	Standard Deviation	Range
Tone	Ability of chatbot to express information in a way that is appropriate for the type of information being delivered.	4	4.25	0.71	3–5	4	4.25	0.71	3–5	4	4.25	0.68	3–5
Clarity	Ability of chatbot to communicate information clearly and in a way that avoids ambiguity or confusion.	4	3.88	1.1	2–5	4	3.75	1.0	2–5	4	3.81	1.05	2–5
Program Accuracy	Ability of chatbot to provide correct information about the In Our DNA SC program	3.5	3.25	1.58	1–5	3.5	3.25	1.28	1–5	3.5	3.25	1.39	1–5
Domain Accuracy	ability of chatbot to provide correct information about the genetic test results and care implications	4	3.88	0.83	2–5	4	3.38	1.06	1–4	4	3.63	0.96	1–5
Robustness	Ability to handle ambiguous queries or incomplete information	4	3.75	0.71	3–5	4	3.88	0.64	3–5	4	3.81	0.66	3–5
Efficiency	Ability to provide answers that are direct, concise, and complete	4	4	1.07	3–5	3.5	3.75	1.16	2–5	3.5	3.88	1.09	2–5
Boundaries	Ability to avoid answering questions that are unrelated to the topic	4	4	0.76	3–5	4	4	0.76	3–5	4	4	0.73	3–5
Usability	Ease of interfacing with Chatbot	4	4.38	0.52	4–5	4	4.13	0.64	3–5	4	4.25	0.58	3–5
	Average Scores	3.92	3.94	0.92	1–5	3.80	3.88	0.91	1–5	3.88	3.86	0.89	1–5

**Table 6:** Description of PGSChat Content

Component of Chatbot	Scripted Content	Open Ended Content
**Overview of PGS Program**	• Reminder of participation in PGS program • Introduction to PGSChat	• Details about specific PGS program
**Return of Positive Results**	• Disclosing results to participant • Overview of what positive results mean • Example of impact of positive results	• Additional examples of the impact of positive results
**Screening Recommendations**	• Screening recommendations for specific mutation	• Additional example of screening recommendations
**Impact on Family and Cascade Testing**	• Importance of informing family members • Summary of cascade testing options	• Additional examples of family history and cascade testing resources
**Next Steps**	• Description of next steps to schedule genetic counseling	• Details about what genetic counseling entails and how to prepare
